# Effective Light Beam Modulation by Chirp IDT on a Suspended LiNbO_3_ Membrane for 3D Holographic Displays

**DOI:** 10.3390/s20041218

**Published:** 2020-02-23

**Authors:** Yongbeom Lee, Keekeun Lee

**Affiliations:** Depart. of Electrical & Computer Eng., Ajou Univ., Suwon, Geonggi-do 16449, Korea; tkrtkrdl1995@ajou.ac.kr

**Keywords:** acousto-optic, waveguide, surface acoustic wave, bragg diffraction, active lens, holographic display

## Abstract

An acousto-optic (AO) holographic display unit based on a suspended waveguide membrane was developed. The AO unit consists of a wide bandwidth chirp interdigital transducer (IDT) on a 20 µm thick suspended crystalline 128° YX LiNbO_3_ membrane, a light blocker with a 20 µm hole near the entrance, and an active lens near the exit. The 20 µm thickness of the floating membrane significantly enhanced surface acoustic wave (SAW) confinement. The light blocker was installed in front of the AO unit to enhance the coupling efficiency of the incident light to the waveguide membrane and to remove perturbations to the photodetector during measurement at the exit region. The active lens was vertically attached to the waveguide sidewall to collect the diffracted beam without loss and to modulate the focal length in free space through the applied voltage. As SAWs were radiated from the IDT, a Bragg grating with periodic refractive indexes was formed along the waveguide membrane. The grating diffracted incident light. The deflection angle and phase, and the intensity of the light beam were controlled by the SAW frequency and input power, respectively. The maximum diffraction efficiency achieved was approximately 90% for a 400 MHz SAW. COMSOL simulation and coupling of mode modeling were performed to optimize design parameters and predict device performance.

## 1. Introduction

Although various types of holographic display technologies are currently available, these displays are not suitable for use in portable devices because of their large volume and power consumption, high cost, and system complexity. For real-time 3-dimensional (3D) holographic video in free space, a spatial light modulator (SLM) is necessary to modulate the intensity, direction, phase, and focal length of the light beam [1,2,3,4]. Most of the current technology in SLMs employs optical addressing technology that either moves the light source directly to form a 3D image or uses a secondary light source with no mechanical movements [5,6]. The weaknesses of these systems include bulky dimension, high power consumption, small bandwidth, blurry images due to small deflection angles, small viewing displays, and poor scalability. Among the possible SLM technologies, acousto-optic (AO) based SLMs are considered to be the most promising candidates for overcoming these limitations as they do not require motors for beam adjustment, and have high light efficiency, scalability, and portability [7,8,9]. Recently, MIT Media Lab reported a promising result for an AO-based SLM [10,11]. Its optical imaging system consists of an AO modulator and a de-scanner. Light illuminating the guided layer was diffracted vertically by surface acoustic waves (SAW) before exiting from the substrate. De-scanning was performed by rotating mirrors. Hologram images were successfully demonstrated in the viewing plane. However, despite these reported achievements for AO-based SLMs, there are still many limitations and challenges for the practical implementation of real-time holographic video displays in free space.

**Figure 1:**
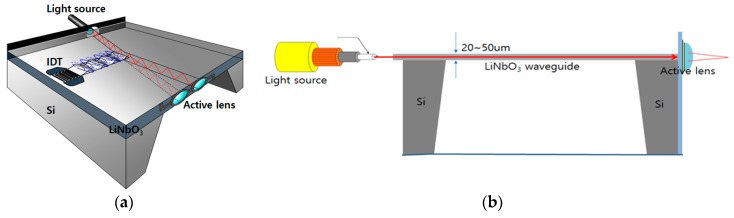
Overall schematic view of the developed AO system. (**a**) 3D view and (**b**) cross-sectional (artificial) view.

To overcome these constraints, we introduce a newly developed SLM unit for 3D holographic display applications. Figure 1 shows the overall schematic view of the AO system. It consists of an optical blocker for effectively coupling the incident light beam to the waveguide layer, a floating waveguide layer surrounded by air, wide bandwidth interdigital transducers (IDTs), and an active lens near the exit of the waveguide layer. An incident beam with a wavelength of 660 nm is coupled to the waveguide layer through the opened hole of the light blocker. Thereafter, the light beam propagates along the waveguide. In the absence of surface acoustic wave (SAW) perturbations, the path of the light beam does not change and is straight. However, the application of a SAW from IDT with wide bandwidth modifies the density of the crystalline 128˚ YX LiNbO_3_ (LN) membrane, causing a change in the refractive index and giving rise to a Bragg grating with a periodic reflective index structure. The incident beam interacts with the traveling SAW and changes its direction when the Bragg conditions are satisfied. Constructive interference by Bragg diffraction forms deflected beam of the first order in which the diffraction efficiency can be up to 100% [12]. The beam exits the waveguide layer and is then focused on the viewing plane by the active lens. The diffraction angle of the incident beam is determined by the distance between the Bragg gratings modulated by the SAW and the glancing angle between the incident beam and the Bragg grating plane. The intensity of the diffracted beam relies on the SAW input power, the IDT aperture length, and the waveguide thickness.

Our SLM unit possesses several advantages over presently existing SLMs. The employment of silicon technology in the AO SLM implementation enables the direct integration of several other electronic components on the same substrate. Another is that in the current AO technology, Ti diffusion method into bulk LN under wet environment is generally used to form a thin waveguide layer with higher refractive index along the top surface of bulk LN [13]. Therefore, it causes several issues such as a difficulty of accurate control of the waveguide thickness and a linearly graded doping profile of Ti along the depth direction of the bulk LN, which means a non-uniformity of the refractive index in the waveguide layer. In contrast, our SLM unit uses a single crystalline LN membrane (n = ~2.21) surrounded by air (n = 1) for the waveguide layer, leading to effective light confinement and reduced light loss. Additionally, no mechanical motors are needed to adjust the focal length of the light beam.

## 2. Optimal Design Consideration and Simulations

**Figure 2:**
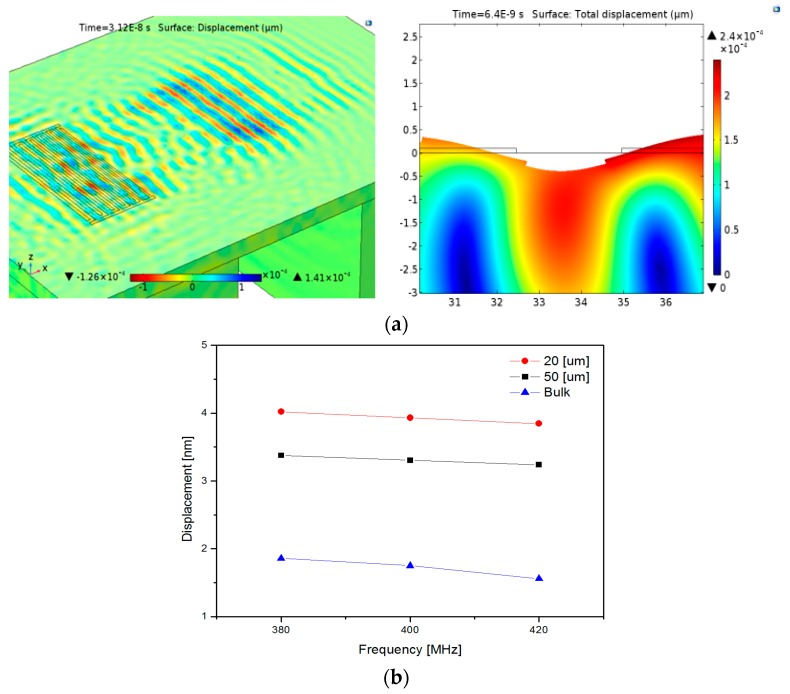
(**a**) Simulated AO unit using COMSOL and (**b**) surface displacement in terms of waveguide thickness and frequency.

COMSOL simulation was performed to predict the effects of the membrane thickness and the etched area underneath the membrane on the device performance in terms of the passband frequencies (Figure 2). A floating LN waveguide membrane supported by bulk silicon at both ends was designed and a chirp IDT was configured on the piezoelectric membrane. Intensive meshes were formed along the LN membrane. The finger periodicity in the chirp IDT was varied gradually to generate a constructive and harmonic SAW with a wide bandwidth between 380 to 420 MHz. A 400 MHz RF pulse was applied to the IDT so that a planar SAW was radiated and propagated along the membrane. This is essential to the formation of a Bragg grating with a periodic refractive index structure. The effects of the membrane thickness and the center frequency on the mechanical deformation near the top surface of the membrane were assessed. A larger elastic strain was observed in the device with a 20 µm thick membrane compared to the device with a 50 µm thick membrane and the device with bulk LN when a SAW with 400 MHz center frequency was applied (Figure 2b). For this reason, we insist that most of the SAW energy is concentrated at a depth equal to one wavelength from the surface. One wavelength of a 400 MHz RF pulse corresponds to 9.7 µm. At this membrane thickness, the bulk loss is decreased, and a harmonic wave between the generated SAW and the wave reflected from the membrane edges are formed. As the membrane thickness increases from 20 µm to 50 µm, smaller elastic strain along the surface was observed. In our previous study [14], when the LN membrane thickness was less than one wavelength, the frequency response at the two port SAW delay line was badly distorted in S_21_. In contrast, the SAW response was greatly improved in case the membrane thickness was slightly larger than a single wavelength about the center frequency [15]. Furthermore, the extremely thin LN membrane was weak and brittle so that it was too difficult for mechanical handling of the device. Additionally, the effect of the etched bulk silicon area on the degree of the strain near the surface was evaluated. A larger deformation along the LN membrane was observed for a device with bulk silicon just below the chirp IDT compared to the device with bulk silicon etched under the IDT. Based on this result, only the silicon underneath the cavity between two IDTs was etched, but the silicon just below the IDT was kept intact in the designed configuration.

**Figure 3:**
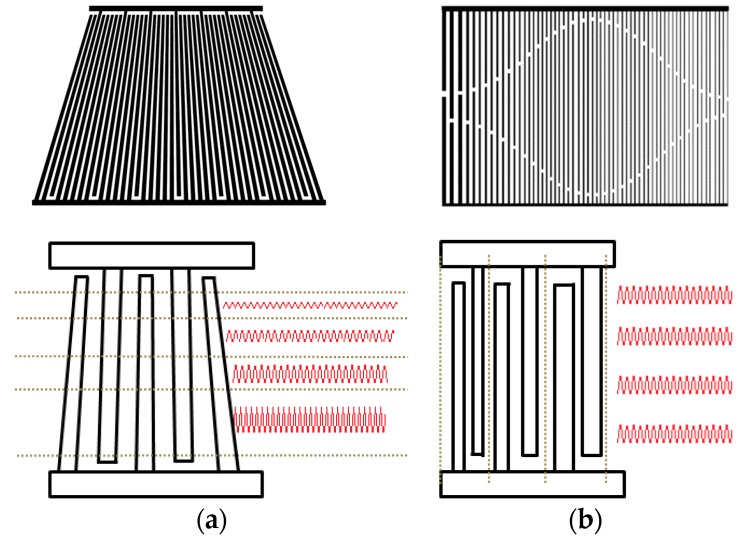
Schematic views of (**a**) slanted IDT (**b**) chirp IDT for COM modeling.

**Figure 4:**
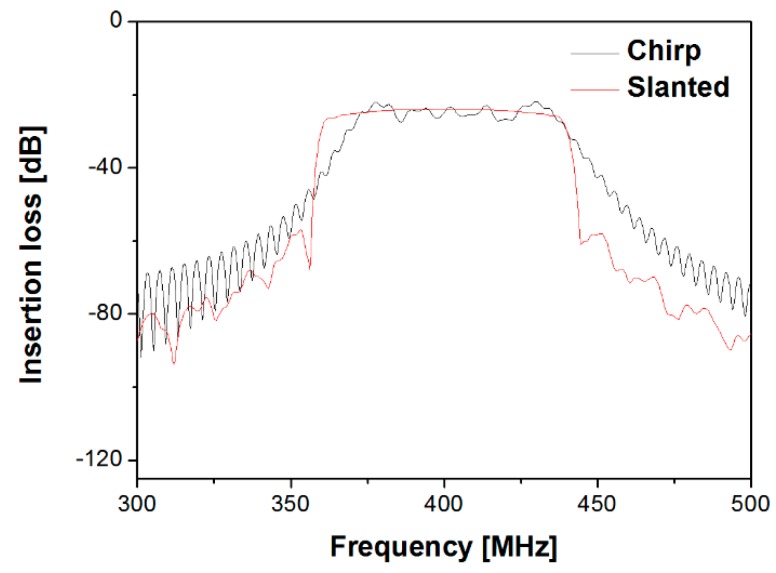
Passband responses of the slanted and chirp IDTs with respect to frequencies obtained from COM modeling.

Coupling of mode (COM) modeling was performed to find optimal parameters with a wide passband bandwidth for slanted and chirp IDTs. A slanted IDT with a 4-degree tilt was designed, as shown in Figure 3a. The IDT is segmented into several stripes in the direction of SAW propagation. Each segment has a different finger period. The structural period at the top segment corresponds to the highest frequency edge in the passband, while the finger period at the bottom segment forms the lowest frequency edge of the passband. Each stripe, consisting of IDTs with small apertures, was modeled with a three-port P matrix and its admittance matrix was calibrated using the general P-matrix method. The detailed procedure of the calibration processes was already presented in the author’s previous articles [16,17,18], so we omit the p-matrix calibration method for finding the admittance matrix and focus on the simulation results here. The frequency responses were obtained by summarizing the admittance matrices of all the stripes. For the chirp modeling, the apodized IDTs with different aperture lengths were configured as shown in Figure 3b. The aperture lengths affect the transducer admittance and impedance matching. The IDTs were segmented periodically along the direction perpendicular to the SAW propagation. The P matrix of each individual segment was calibrated. Next, all the P matrices were cascaded to obtain the total admittance of the entire IDT period. The smallest period in the IDT determines the high frequency edge of the passband, while the largest period corresponds to the low frequency edge. Table 1 shows all the COM parameters used in the modeling. Figure 4 shows the passband responses of the slanted and chirp IDTs in terms of the frequencies obtained by the COM modeling. A wide bandwidth and a steep sidelobe were observed from the designed transducers. The frequency response in the passband region was uniform within a wide frequency range by taking into account the different aperture lengths in the chirp IDT, appropriate tilt angle in the slanted IDT appropriately, and matched impedance with frequency in the modeling. Based on the obtained parameters, the transducers were designed and fabricated.

## 3. Experimental Methods

**Figure 5:**
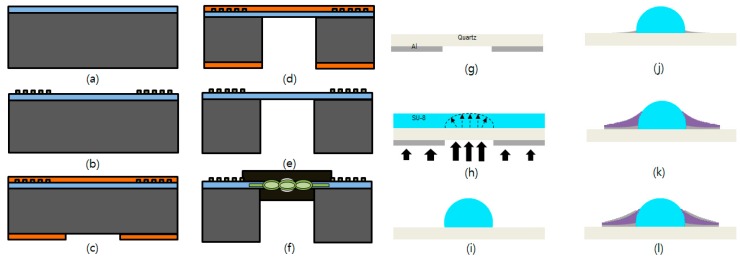
Fabrication procedure. (**a**) LN waveguide bonding onto Si wafer, (**b**) Al patterns for wide bandwidth IDTs, (**c**) PR patterns for masking and protection layer during DRIE process, (**d**) DRIE, (**e**) cleaning with microstriper, (**f**) light blocker assembling for effective light coupling, (**g**) Al patterns for lens, (**h**) SU-8 spin coating and then UV exposure via proximity lithography, (**i**) developing process to form hemisphere shape of the polymer, (**j**) Al patterns, (**k**) PZT patterns via RF sputter, and (**l**) Al patterns for sandwiched PZT by two electrodes and lift-off.

Two wafers, 4″ LiNbO_3_ and silicon, were bonded together and then treated by chemical mechanical polishing (CMP) to reach to the desired thickness of the piezo membrane, which was processed by Nanoln (Jinan Jing zheng Electronics Co.) on customer demand (Figure 5a). A 128° YX LiNbO_3_ was chosen as the top piezoelectric membrane because it provides a Rayleigh wave, a high SAW propagation velocity, and a large electromechanical coupling factor [19,20,21]. A 1 µm thick photoresist (PR) layer was spin-coated and patterned for the slanted and chirp IDTs, followed by a 200 nm thick aluminum deposition via a thermal evaporator and lift-off by a microstriper (AZ400T) (Figure 5b). After completing the IDT patterns on the top surface, a 20 µm thick PR (AZ 4620) layer was patterned on the back side of the wafer as a masking layer for the subsequent deep reactive ion etch (DRIE) process in which the top surface was protected with PR as well (Figure 5c). Next, 500 µm of bulk silicon was etched away by DRIE until only the floating LN membrane was left [14,22,23]. Etch time and etch rate were carefully monitored and controlled. All the silicon substrate was etched, leaving only the top LN membrane (Figure 5d). The bulk silicon near the entrance zone of the unit was kept intact to provide mechanical strength for easy handling, as shown in schematic of Figure 1a. The etched wafer was then dice-sawed into individual completed devices. Final mechanical polishing was performed on a fine polishing pad manually to eliminate any microroughnesses around the side walls of the LN waveguide membrane to minimize scattering and spreading of the exiting light beam. Afterward, a 20 µm open hole on a light shielding film was made by using the same method as the process of making a film mask to form an optical blocker at the entrance region (Figure 5f), which was processed by Nepco Co. on customer demand. An optical coupling liquid matching layer was applied to the sidewall of the LN membrane at the exit to maximize the intensity of the beam exiting from the waveguide membrane. 

For the active lens, aluminum was deposited and then patterned on a 200 µm thick 4″ quartz wafer (Figure 5g). On the other side of the quartz wafer, a thick SU-8 polymer layer was spin-coated and then UV-exposed from the side on which aluminum was patterned (Figure 5h). The proximity contact method was used in the UV exposure process to form a hemispherical shape on the negative SU-8 polymer (Figure 5i). After the developing process, a 300 nm thick aluminum was deposited using a thermal evaporator and then patterned around the lower part of the hemisphere (Figure 5j). Next, PR was spin-coated and then patterned for subsequent lift-off process, followed by a 1 µm PZT deposition in the sputter system and a 300 nm aluminum deposition in the e-beam evaporator and then the PR dissolving in acetone (Figure 5l). The PR was not damaged during the sputtering process, and was dissolved well in acetone [22,24,25]. The PZT was sandwiched by two aluminum electrodes around the entire circumference of the polymer lens. Finally, the lens on the quartz plate was dice-sawed to fit the exit facet of the waveguide membrane, then attached to the side wall of the fabricated AO unit.

## 4. Results

### 4.1. Fabricated Devices

**Figure 6:**
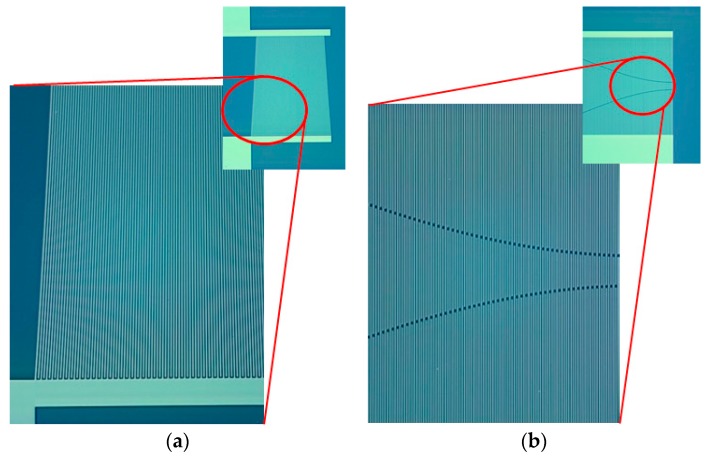

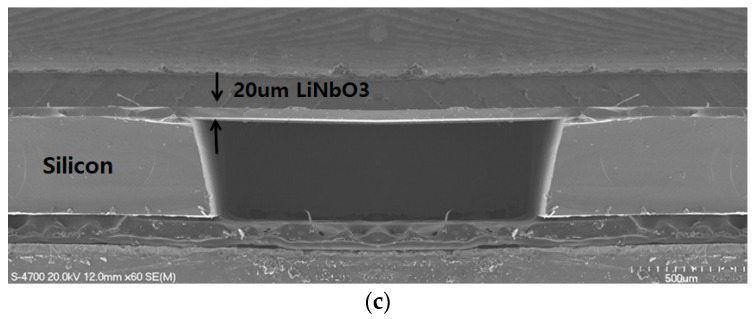
Optimal images of the fabricated (**a**) slanted and (**b**) chirp IDTs. (**c**) Cross-sectional view of the LN membrane via SEM.

Figure 6 shows optical images of the fabricated IDTs. Two different types of IDTs, slanted and chirp, were developed. For the slanted IDTs, the number of finger pairs, the aperture size, and the tilt angle with respect to the normal to the SAW propagation axis are 120, 0.388 mm, and 4°, respectively. The maximum and minimum finger widths of the slanted IDTs are 2.6 μm and 2.2 μm, respectively, and the aluminum thickness is approximately 150 nm. For the chirp IDTs, the number of finger pairs is 200, and the aluminum thickness of the IDTs is the same as that of the slanted IDTs. Figure 6 shows the cross-sectional view of the floating LN membrane. A 20 μm thick LN waveguide layer is surrounded by air. Several waveguide thicknesses in the range of 20 to 50 µm were tested to find the optimal thickness for device performance in terms of the passband frequency. The distance between the inlet and the outlet of the waveguide layer was ~6.7 mm. The width of the device was large enough to prevent any SAW reflection from the edge. For efficient incident beam coupling into the LN waveguide membrane, a light shielding plate with a 20 µm diameter open hole was attached tightly in front of the system. The light shielding plate also prevents interference of the light beam at the photodetector near the exit region. An active lens of 10 µm height was fabricated from SU-8 polymer on quartz and then put together at the exit of the system. 

### 4.2. IDT Characterization

**Figure 7:**
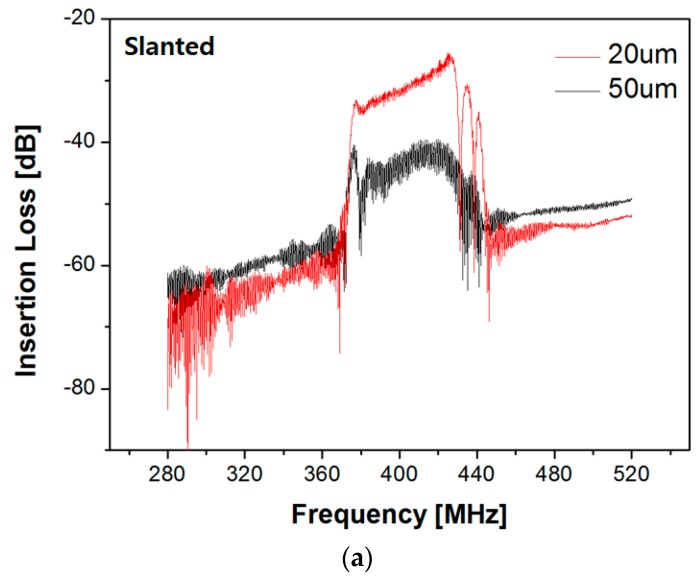

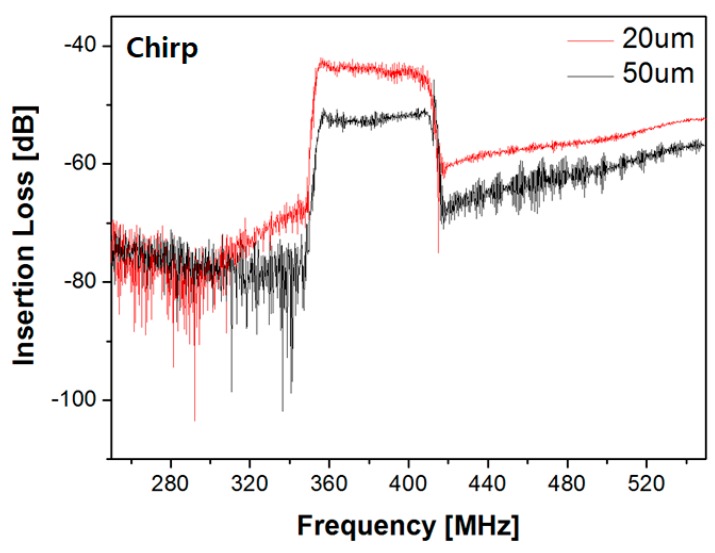
Experimental insertion loss (S_21_) vs. frequency in terms of LN thicknesses from (**a**) slanted IDT and (**b**) chirp IDT.

First, slanted IDTs on a 20 µm thick LN membrane were characterized by a network analyzer at room temperature. The delay distance between input and output IDTs was approximately 3.5 mm. A wide bandwidth of 370–430 MHz with a ~30 dB sidelobe was observed in S_21_ measurement (Figure 7a). The lower insertion loss was observed from the IDTs on 20 µm thick membrane compared to the result on a 50 µm membrane. We attribute this to the same reason mentioned in the COMSOL simulation section; i.e., the SAW energy is more concentrated in the membrane with 20 μm thickness and the energy loss to the bulk is greatly reduced. The chirp IDTs were also characterized under the same conditions. As shown in Figure 7b, a wide bandwidth of 360–420 MHz was observed in which the bandpass was centered around 380 MHz. The insertion loss in the bandpass region was approximately ~42 dB. The slanted IDTs showed somewhat better performance over the chirp IDTs in aspect to the insertion loss because each segment in the frequency response along the aperture length of the slanted IDT works separately, not perturbing the responses in other segments significantly. In contrast, the chirp IDTs are strongly related with one another when they form a harmonic wave in frequency response. This makes it difficult to form harmonic waves as they pass through hundreds of IDT pairs, thus resulting in greater insertion loss in the passband. However, for a higher diffraction efficiency in the AO SLM unit, a larger interaction region is required between the incident light beam and SAW energy. The slanted IDT radiates the SAW of a specific frequency from only a small segment in IDT, whereas, the chirp IDT emits the SAW from entire IDT aperture, providing a larger interaction region between the incident light beam and acoustic energy. For higher diffraction efficiency, the chirp IDT might be a better solution in principle.

### 4.3. Light Coupling with Waveguide Membrane

**Figure 8:**
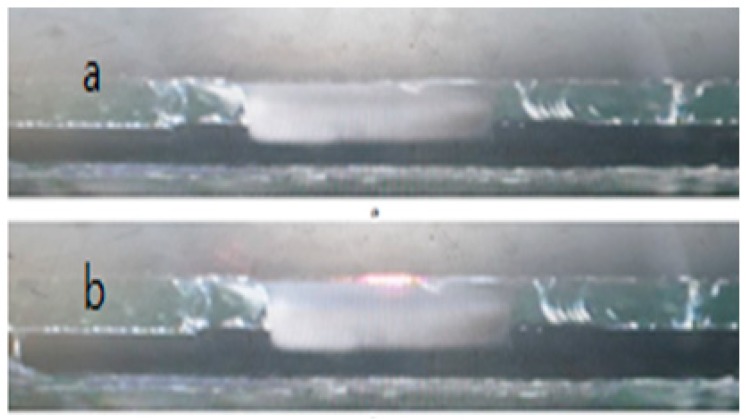
(**a**) Exit view from CCD camera without incident light beam (**b**) the zeroth order light beam at exit without SAW perturbation.

The waveguide was illuminated by 660 nm wavelength light through an open hole of the light blocker. Without any SAW perturbations from the chirp IDT, the light beam propagated in a straight direction, resulting in a finite zeroth order intensity at a photodetector near the exit (Figure 8). The incident beam was well confined in the waveguide layer and propagated along the layer because of the higher refractive index of the waveguide compared to the surrounding layer and the uniform doping profile along the entire waveguide membrane region. Further, the ~4 eV energy bandgap of the LN membrane at 300 K prevented any optical absorption of the propagating light beam when the light traveled along the waveguide membrane [26,27]. To maximize the intensity of the exiting beam, a 1/4 wavelength-thick optical coupling liquid was applied to the side wall of the waveguide membrane for optical impedance matching, and attempts were made to ensure that the walls were smooth and damage-free. Nevertheless, the propagating light beam was a little bit scattered and dispersed when it exited out of the LN waveguide crystal, as shown in Figure 8b.

### 4.4. Changes of Direction, Intensity, and Phase of the Light Beam via AO Interactions

**Figure 9:**
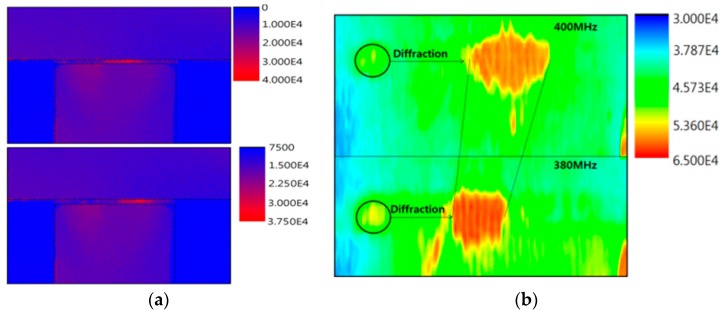

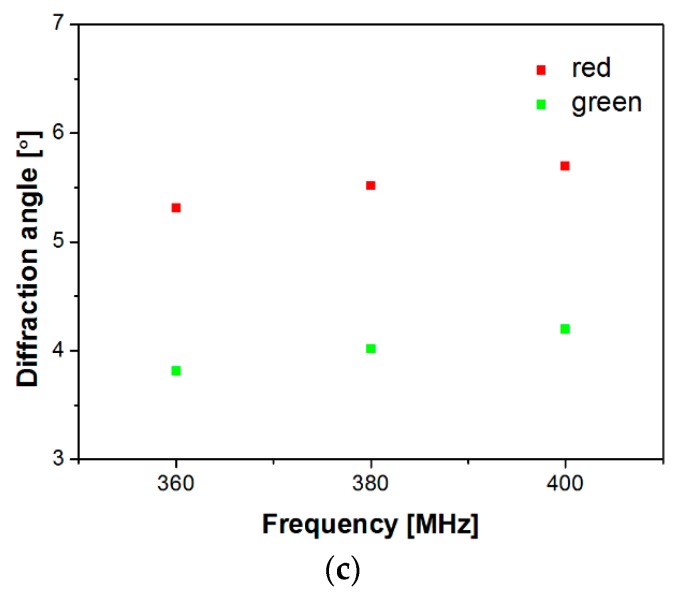
(**a**) CCD views by applying different frequencies from chirp IDTs. (**b**) Contour plot and color mapping with respect to different SAW frequencies. (**c**) Diffraction angle vs. SAW frequency in terms of the wavelength of the incident light beam.

**Figure 10:**
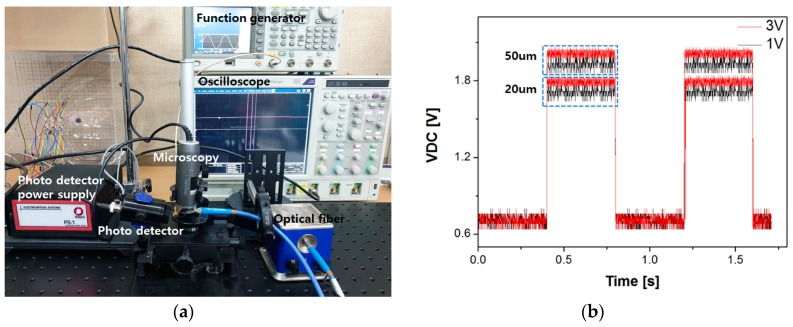
(**a**) Measurement setup for the diffraction efficiency after placing a photodetector at the first order beam position and (**b**) output voltage of photodetector before and after beam diffraction.

A 400 MHz SAW was applied by the chirp IDT at a glancing angle to the incident beam. The SAW formed a Bragg grating with a periodic refractive index according to the applied SAW frequency. The direction, intensity, and phase of the incident light were changed by the interaction with the SAW acoustic energy [28]. As shown in Figure 9, the incident light was diffracted ~5.5˚ away from the zeroth order axis when a SAW with a fixed frequency of 400 MHz was applied. Different SAW frequencies were applied by the chirp IDT, resulting in different deflection angles as shown in Figure 9c. Smaller deflection angles were observed as the applied SAW frequency decreased, in good agreement with the Bragg law. Next, the wavelength of the incident beam was changed from red to green range. As shown in Figure 9c, the shorter the wavelength of the incident beam, the smaller the deflection angle. The SAW input voltage for the 400 MHz SAW generation was changed from 1 V to 3 V in the peak-to-peak using a high frequency signal generator. Any heat-related problem by SAW generation was not observed in the applied voltage range. For a quantitative analysis, a photodetector was installed near the exit to compare the intensity changes between the zeroth order and the first order beam (Figure 10a). The diffraction efficiency reached as high as ~90% for a 400 MHz SAW. Generally, the diffraction is defined as the ratio between the diffracted beam intensity at the exit of the waveguide layer and the incident light beam intensity. In the other way, the diffraction efficiency can be simply defined by comparing the undiffracted and diffracted light densities driven by SAW. We selected latter way to monitor the diffraction efficiency. The evaluated efficiency was ~90% when a SAW of 400 MHz was applied. This diffraction efficiency varies depending on the SAW input power even at the same frequency. As the input voltage in the peak-to-peak voltage was increased, a larger output light intensity was observed at the first order position due to a stronger AO interaction (Figure 10b). Larger SAW input power increases the interaction energy between AOs, thus leading to higher diffraction efficiency. The response and recovery time of the diffracted beam were examined when the SAW was applied from the chirp IDT. As soon as SAW was applied, the diffraction occurred almost instantaneously. Figure 10b shows the response time of the diffracted beam when SAW input power turns on and off, in which the measurement was fulfilled by placing photodetector at the position of the first order spot at the exit. As shown in Figure 10b, the intensity of the diffracted light beam at the first order spot position was significantly increased in a moment when the SAW was radiated. No delay time was observed, proving that the response time of the diffracted beam due to SAW radiation is fast enough for the AO device to form video-quality image. The efficiency for different waveguide thicknesses was then compared. As predicted, as the thickness became close to one wavelength of the passband frequency like a 20 µm thick membrane, the diffraction efficiency became higher because of the better confinement of SAW energy across the 20 µm thick membrane compared to thicker membranes (Figure 10b). Detailed reasons regard this result were already explained in optimal design consideration and simulations section, so we omit the specific discussion of the results.

### 4.5. Active Lens and Image Formation Process

**Figure 11:**
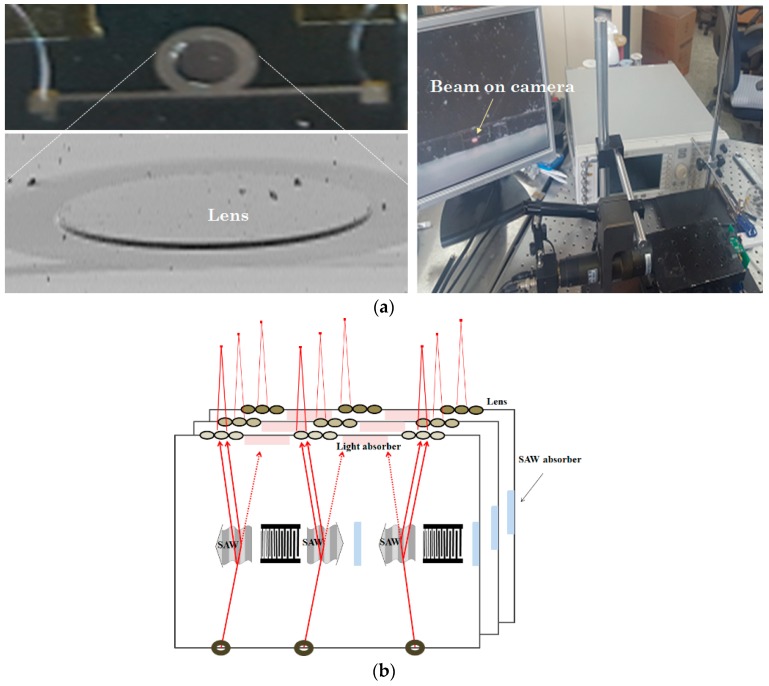
(**a**) Optical and magnified views for active lens testing and (**b**) visual view for AO stacks and lens array to form holographic video.

An active lens was installed at the exit zone to modulate the focal length of the exit beam and form an image pixel at a certain distance in free space (Figure 11a). For quantitative analysis of the active lens, we placed a single photodetector at a certain distance from the exit of the waveguide layer and compared the V_out_ of the photodetector in case with and without the lens. A ~10% increase in V_out_ of the photodetector was observed when the lens was installed because the spread light was collected at a single point toward the detector. When cyclic voltage of 8 V was applied to the aluminum electrodes sandwiching the PZT under a 400 MHz SAW perturbation, a noticeable change in the focused beam diameter on charge-coupled device (CCD) camera placed at a fixed distance from the lens was observed. This indicates that the PZT strain near the lower region of the hemisphere contributed to the change in lens shape. Further increase in applied peak-to-peak voltage in active lens more widely disturbed the focused beam diameter on the CCD camera. The use of arrays of such active lenses for modulating the focal length within a cube-like volume matrix in free space can eliminate the need for motors for beam conditioning (Figure 11b).

## 5. Conclusions

We developed a highly efficient diffractor based on a suspended LiNbO_3_ waveguide layer and a wide bandwidth chirp IDT for 3D holographic displays. The use of the thin LN membrane as the waveguide layer enhanced the confinement of SAW energy within waveguide, leading to increased diffraction efficiency and coupling efficiency into the membrane. An active lens was also installed onto the sidewall of the exit region to form a focused beam at a certain distance in free space. The position of the focal point, the direction of the diffracted beam, and the beam intensity were successfully modulated by the applied voltage to the active lens, the SAW frequency, and the SAW input power, respectively. The response time of the deflected beam when the SAW was turned on and off was fast enough for the AO device to form video-quality images. The light beam propagating along the waveguide layer was deflected by 5.5° using a 400 MHz SAW grating and imaged on the plane. Array and stacked configurations of AO units and the integration of active lenses will be the focus of future work.

## Figures and Tables

**Table 1:** COM parameters used in the modeling.

Velocity [m/s]	3880.5
Capacitance [F]	40 × 8.85 × 10^−12^
Electromechanical Coupling Factor	0.04
Strength of Reflective per Finger: K1	−0.015
Reflectance [%]	2 × K1
Number of Input IDT: N	100
